# National Creutzfeldt–Jakob disease research biobank, a novel approach to the establishment of the scientific platform: collaboration between patient advocacy group, scientists, regulators and physicians

**DOI:** 10.1186/s13023-025-03703-6

**Published:** 2025-04-10

**Authors:** Alice Anane, Doron Pasternak, Shimon A. Reisner, Victor Novack

**Affiliations:** 1 Creutzfeldt–Jakob Israel Foundation, Pardes Hanna, Israel; 2 Negev BioBank and Clinical Research Center, Faculty of Health Sciences, Soroka University Medical Center, Ben-Gurion University of the Negev, PO Box 151, Be’er-Sheva, 84101 Israel; 3 Israeli National BioBank for Research (MIDGAM), Rechovot, Israel

**Keywords:** Creutzfeldt–Jakob disease, Prion diseases, Genetic Creutzfeldt–Jakob disease, Gerstmann–sträussler–scheinker disease, Fatal familial insomnia, Genetics, Prion proteins, PRNP, Biobank

## Abstract

Creutzfeldt–Jakob disease (CJD) is a severe neurodegenerative disorder characterized by the abnormal accumulation of prion proteins. In Israel, a unique epidemiological pattern of CJD has been identified, specifically a genetic form (gCJD) associated with the E200K mutation in the PRNP gene. Investigating rare diseases such as CJD syndrome poses challenges due to their low prevalence, hindering the formation of an adequate patient cohort for comprehensive research and treatment trials. To overcome this limitation, biobanks have emerged as transformative tools for collecting and distributing biological specimens along with corresponding health data. Biobanks offer a solution to the inherent heterogeneity in rare diseases, allowing researchers to access diverse and extensive sample sets, thereby enhancing the understanding of disease nuances toward potential therapy. We introduce a novel collaborative model involving the Negev BioBank (NBB), the Creutzfeldt–Jakob Israel Foundation, the Israeli National BioBank for Research (MIDGAM), and the Israeli Ministry of Health. Each entity contributes unique expertise and resources to establish a comprehensive platform for studying the disease. The goal was to establish a participant pool of 500 individuals, including clinically diagnosed cases, confirmed carriers of the E200K mutation, and their first- and second-degree relatives. During the pilot phase of the last year, 250 participants were enrolled, with each family contributing between 1 and 25 participants. This collaborative approach involving communities, scientists, physicians, and regulatory bodies establishes a model applicable across various fields. These synergistic efforts aim to advance research on CJD and potentially serve as a blueprint for studying other rare diseases.

**Supplementary Information:**

The online version contains supplementary material available at 10.1186/s13023-025-03703-6.

## Introduction

Creutzfeldt–Jakob disease (CJD) is a rare devastating neurodegenerative disease characterized by the pathological aggregation of prion proteins and is classified as part of a group of prion diseases. Its rapid progression leads to severe neurological debilitation, invariably resulting in death. CJD has an annual incidence of 1–2 new cases per million individuals worldwide [1]. European countries report varying incidence rates of 1.4 cases per million people, while Slovakia has a high incidence in certain regions [2, 3]. These figures underscore the disease’s global impact but reveal regional variations in its occurrence. The majority of prion diseases are sporadic, accounting for approximately 85% of cases [1]. Genetic forms including genetic CJD (gCJD), Gerstmann–Sträussler–Scheinker syndrome (GSS), and fatal familial insomnia (FFI), cause 10–15% of cases. Over 60 rare variants in the PRNP gene have been reported to cause genetic prion diseases [4], among which gCJD with the mutation E200K is the most prevalent [5].

E200K is common within specific communities, notably those of Libyan and Tunisian Jews [6] and individuals of Italian [7] and Slovakian descent [8].In Israel, there are large communities of Libyan and Tunisian origin Jews, creating an ideal conditions for establishing a longitudinal biobank for gCJD in Israel.

**Fig. 1:**
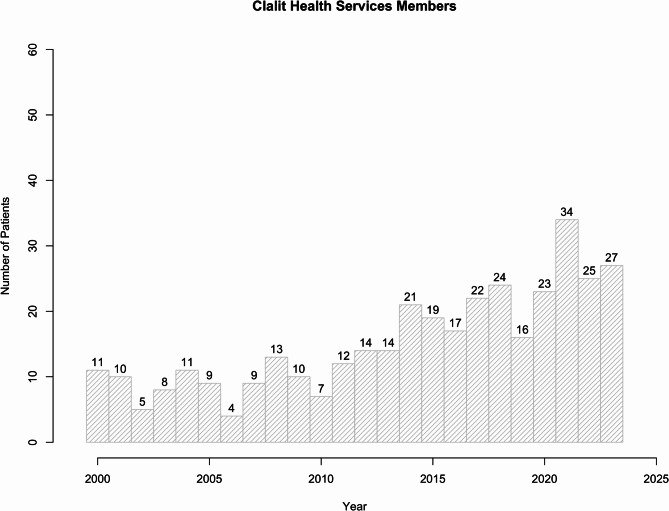
Annual distribution of new CJD cases in Israel among Clalit Health Services members

Clalit Health Services is the nation’s largest Health Maintenance Organization (HMO) in Israel, serving more than 4.7 million individuals, comprising 52.3% of the entire population. This HMO has offered a comprehensive range of healthcare services for more than a century, and approximately diagnoses around 25–30 new CJD cases annually according to recent data (Fig. 1). These figures can be extrapolated to the national level yielding an estimate of 50 new cases diagnosed yearly. When sporadic variants are excluded, it can be estimated that approximately 40 of these cases are due to genetic factors, which translates to a national annual incidence rate of gCJD of approximately 1 in 250,000. The observed incidence rates, especially for the genetic variants of the disease, significantly diverge from global averages, emphasizing the role of genetic factors in the incidence of CJD in specific populations.

Research on rare diseases such as CJD presents unique challenges. Due to their low prevalence, gathering a sufficient patient pool becomes a significant limitation crucial for understanding disease progression and determining disease treatment potential [9]. Additionally, many rare diseases exhibit high heterogeneity, leading to a lack of standardized diagnostic criteria and treatment approaches, factors detrimental to research with small sample sizes [10]. Furthermore, the highly variable age of disease onset in genetic forms of CJD poses a significant challenge for designing clinical trials, as it complicates the identification of appropriate time windows for therapeutic intervention and makes it difficult to predict when individuals might benefit most from preventive treatments [11]. Such limitations can deter pharmaceutical companies from investing in developing treatments for rare diseases due to the smaller potential market size, not justifying the research and development costs.

Biobanks have emerged as transformative tools in the scientific landscape. Biobanks collect, store, and distribute biological specimens and their corresponding health data. The inherent heterogeneity of rare diseases, which complicates the research process, can be more effectively addressed through the use of diverse and broad-scale sample sets from biobanks. In longitudinal research on specimens’ cohort, researchers can gain insights into the disease spectrum, facilitating a deeper understanding of its nuances. This extensive repository allows for a more detailed analysis of disease variations, aiding in differentiating standard patterns from outliers. As a result, biobanks not only enhance the potential for more precise diagnostic tools, biomarkers and early diagnosis but also pave the way for the development of tailored therapeutic interventions, pushing the boundaries of what is achievable in rare disease research.

### The importance of researcher-community engagement in global biobanks

The efficacy and success of a biobank’s research efforts greatly depend on its relationship with the targeted participant community. Consistent and transparent engagement can build trust, enhance data quality, and improve participant preservation.

One of the prominent features of the UK Biobank, among the largest and most detailed biobanks in the world, is its community feedback mechanism. The use of a mix of communication channels such as emails, newsletters, and social media platforms keeps participants informed and allows them to feel valued and involved. This communication approach ensures that participants stay updated about how their contributions are utilized and the progress made on various research fronts. As a result, participants are more likely to remain engaged in the long term, ensuring a stable and up-to-date database for researchers [12]. In the USA the Precision Medicine Initiative’s “All of Us” program pioneered an interactive, online engagement model. Participants are not just passive donors; they have real-time access to their data and can see how it shapes research [13]. The RD-Connect Registry & Biobank Finder integrates various rare disease biobanks and is a centralized hub connecting biobanks, databases, and patient registries. By offering unified access to diverse resources, it accelerates research and fosters collaboration between researchers and patient communities. This integrated model ensures that patients with rare diseases can directly influence research related to their conditions [14].

Another significant initiative is Global Genes, a leading rare disease patient advocacy organization seeking to connect, empower, and inspire the community. Through collaboration, education, and the sharing of resources, they combat the challenges of rare diseases. By fostering global collaboration and hosting events, they bridge the gap between affected individuals and researchers, ensuring that research aligns with patient needs and concerns [15].

In line with these established engagement principles, our biobank maintains regular communication with the CJD community through the Creutzfeldt-Jakob Foundation’s newsletters, which keep participants informed about research progress and developments. As our platform develops, we plan to expand these engagement channels to include additional feedback mechanisms and data access tools. The Foundation’s established presence and deep community trust provide a strong foundation for these engagement efforts, enabling effective communication between researchers and families affected by CJD.

Drawing insights from these examples, it is evident that when biobanks prioritize and innovate their engagement strategies, they set the stage for a symbiotic relationship with their communities. Such engagement ensures data richness and promotes a sense of collective ownership and purpose, driving the biobank and its participants toward a common goal of advancing medical research.

### Novel model of collaboration

Addressing the profound challenges in CJD research necessitates a strategic and collaborative approach. Traditional CJD surveillance centers and biobanks typically operate within defined national boundaries, with limited sample sharing. Disease-specific biobanks like the Progeria Research Foundation Cell & Tissue Bank and the Friedrich’s Ataxia Research Alliance (FARA) biobank have established important frameworks for rare disease research [16, 17]. While FARA actively engages with regulatory agencies for drug development and clinical trials, our model is distinctive in incorporating the regulator as a direct collaborative partner in biobank operations. These biobanks share several foundational elements with our approach: they maintain close collaboration with patient advocacy groups, collect samples from both affected individuals and family members, and follow standardized protocols for sample collection and storage. Our platform builds upon these established approaches while introducing distinct elements through integration with a comprehensive healthcare system, enabling access to complete longitudinal clinical histories alongside biological samples.

Here, we present a unique collaboration model between the Negev BioBank (NBB), Clalit Health Services, the Creutzfeldt–Jakob Foundation in Israel, the Israeli National BioBank for Research (MIDGAM), and the Israeli Ministry of Health, each contributing distinct expertise and resources to the collective research of the CJD.

The NBB is a joint venture of Soroka University Medical Center (SUMC), the 1200 tertiary teaching hospital belonging to Clalit Health Services, and Ben-Gurion University of the Negev, a major research university. The NBB combines advanced scientific capabilities with large-scale population and disease-specific collections. By utilizing the integrated healthcare system of SUMC and Clalit, the NBB offers researchers access to participants’ data including their natural history.

The Creutzfeldt–Jakob Foundation was founded in 2008 by Mrs. Alice Anane. After discovering her and her daughter’s carrier status for genetic CJD, Mrs. Anane has been committed to developing a research model that deepens the understanding of CJD triggers and biomarkers. The foundation aims to build a robust data and insight platform by engaging with over 500 families in the association registry, leading researchers and industry to achieve a comprehensive understanding of CJD.

MIDGAM - The Israeli National Biobank for Research was established by Ministry of Health as an infrastructure for the advancement of biomedical research and industry in Israel and for international cooperation. The MIDGAM incorporates biobanks in a number of hospitals in Israel and leads at the national level in providing solutions to professional and ethical problems in the field of biobanking.

The Israeli Ministry of Health provides regulatory oversight and support, ensuring ethical and regulatory compliance and aligning with national health objectives. This support offers a stable and organized framework that enables the development of a collaborative model and paves an ethical and conducive path to innovative research.

**Fig. 2:**
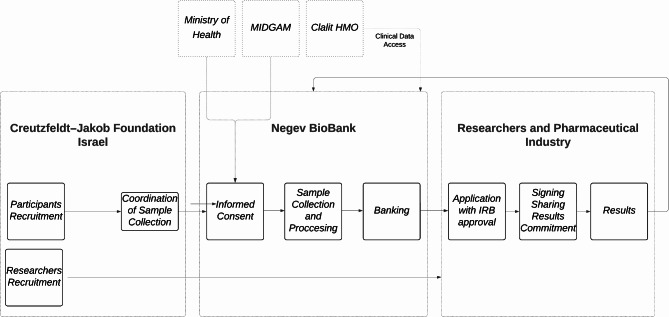
The collaborative operational model of the CJD BioBank. MIDGAM - The Israeli National Biobank for Research

In a pioneering effort to enhance research in the CJD, the NBB consolidated a collaborative partnership with the Creutzfeldt–Jakob Foundation Israel to establish a biobank platform for CJD research. The novel operational model illustrated in Fig. 2 transforms traditional biobank practices through several innovative approaches.

The model implements a distinctive dual-engagement strategy that actively cultivates supply and demand– recruiting participants through the patient advocacy group while engaging researchers worldwide. This focused approach, particularly within Israel’s population having the world’s highest prevalence of gCJD, enables achieving meaningful sample sizes that would be impossible in general biobanks or regions with lower disease prevalence.

Central to this model is a reciprocal value system where researchers receiving samples must share their findings with the biobank, creating a cumulative knowledge base for each sample. This approach generates multiple data points from various researchers, minimizing bias and deepening our understanding of each case.

The community, researchers, physicians, industry, and regulatory bodies have united to form a comprehensive platform integrating diverse data streams - from biological sample analyses to clinical records and environmental questionnaires. This integration is achieved through a sophisticated federated information system built on secure cloud infrastructure, which enables secure data sharing and analysis across multiple research groups. Our platform creates virtual research environments where findings can be shared and analyzed collectively. This integrated approach enables sophisticated meta-analyses and machine learning applications that synthesize diverse data types - including demographic information, biological sample analyses from various researchers, clinical records, and environmental data. According to this model, each sample will have a cluster of cumulative values, resulting in minimal bias and high standardization. This synergistic approach ensures that the resources and findings are continually shared, integrated, and built upon, creating a dynamic research ecosystem.

Our federated data system creates a comprehensive research ecosystem that facilitates collaboration between sample collection, research, and data analysis. Most distinctively, this system integrates three layers of information: biological samples and their research findings, complete longitudinal medical histories from the healthcare system, and self-reported environmental and lifestyle data from participants. This multi-layered integration enables unprecedented insights into disease progression and potential triggers.

### Biobank objectives

1. Establishing a Knowledge Sharing Platform: Users will be required to share their research findings with the biobank on each sample, fostering the exchange of research concepts.
2. Development of an inclusive data repository encompassing clinical, biological and environmental information for each participant:
  - Clinical data records and natural history of the participants sourced from electronic medical records of Clalit HMO, and potentially other HMOs in the future.
  - Biological information obtained from the research results.
  - Environmental information from questionnaires on well-being and lifestyle.

3. Promoting Academic Research: The biobank strives to create opportunities for further research through collaborations with eminent scientists worldwide, targeting a comprehensive multisystem approach, biomarkers, triggers, incubation time, early diagnosis, identification of therapeutic targets, and prevention.
4. Facilitating biomarker and drug development initiatives in the biotech and pharma sectors: The biobank will engage with the pharmaceutical and biotechnology industries to facilitate and endorse collaborative projects between academia and industry.

### Ethical considerations

A significant hurdle for biobanks involves potential participants fearing the implications of discovering that they carry the gCJD gene. Additional concerns stem from concerns about the revelation of their genetic status to others. Despite the broader benefits of this research, many individuals are hesitant due to potential emotional and social consequences. Notably, 95% of participants opted to remain uninformed about their genetic status based on our observations. Nevertheless, we advise those interested in obtaining results to pursue a formal genetic consultation, which can be performed under the current health basket at no charge.

### Enrolment

To encourage enrolment, the Creutzfeldt–Jakob Foundation Israel has also partnered with an organization that represents descendants of Libyan Jews. Interested individuals can participate in the research program by registering through the organization’s website. During registration, participants provided information on their diagnosis status and willingness to provide various samples for research purposes. After registration, the Foundation processes the data and plans the next stage of samples acquisition. This organized approach ensures that the collection process is efficient and accessible to all willing participants.

The comprehensive enrolment process established a diverse and extensive participant pool and fostered a sense of community and shared purpose among those affected by Creutzfeldt–Jakob disease.

### Sampling protocol and storage

The quality of biological samples is critical for research studies. In this regard, we have implemented a rigorous protocol for collecting, handling, and storing biological samples, which ensures the highest quality samples for researchers.

A specially trained NBB team guides participants through the sample collection phase, which follows strict protocols to maintain the integrity of the collected samples. Before collecting any samples, participants were required to provide informed consent using MIDGAM’s comprehensive consent form (see Appendix A).

The trained nursing staff supervised the collection of urine, swabs, and blood samples from each participant. Other samples will also be collected in the future, according to the researchers’ requests. After collection, the samples were promptly transported back to NBB for processing. The samples were processed within four hours of collection, with all procedures being executed at SUMC. Blood samples are further processed into serum, plasma, peripheral blood mononuclear cells (PBMCs), and whole blood, while urine and swab samples are directly frozen. These samples were then promptly frozen and stored in dedicated freezers at -80 degrees Celsius. This temperature is rigorously maintained to ensure the long-term preservation of the samples until they are requested for shipment by researchers, both locally and internationally.

The participant records at the NBB are coded to preserve patient identity, carried out and kept exclusively by the NBB, and not shared with any other party. This coding was intended to maintain confidentiality and the identity of the participants. In this way, the identity of the participants will not be passed on to the research partners, except for the party/person responsible for identifying the participants at the NBB. Furthermore, to maintain privacy, only the birth year of participants is displayed, excluding specific dates.

When researchers request samples for their studies, the NBB team prepares them for shipment. Each consignment involved thorough packaging, incorporating dry ice to sustain the samples at the necessary freezing temperature during transportation. This rigorous procedure is employed to maintain the quality and integrity of the samples throughout the shipping process.

### Governance and sharing of samples

The CJD biobank is owned and managed by a steering committee, in a unique model, equally shared by the NBB and the community represented by the association Creutzfeldt–Jakob Foundation Israel. The Creutzfeldt–Jakob Foundation Israel Foundation works to establish connections with researchers and welcomes researchers to discuss potential collaborations. Once a joint discussion has taken place and approval has been granted by both the steering and the professional committees, the research applies to the local IRB committee or a supreme committee of the Health Ministry for international or genetic research.

The MIDGAM scientific advisor accompanies and supports the applications to the Supreme Committee for ethical approval. After obtaining ethical approval and signing a data-sharing document, we authorized the release of samples to a specific researcher.

Our default policy is to provide support to the researcher if she or he does not have adequate funding to cover the cost of the samples. We request that the obtained results be shared with the CJD biobank within one year for unlimited use to foster future research programs.

### Participants overview

In the project’s pilot stage, we established and optimized procedural standards. Table 1 presents the demographic distribution across the ages and sexes of the 250 participants. These participants included families from Israel, with each family enrolling between 1 and 25 participants. Kinship was classified into three degrees of closeness: first, second, and third. The first-degree includes immediate family members such as siblings, parents, and children; the second-degree comprises grandchildren, grandparents, aunts, and uncles; and the third-degree includes nieces, nephews, second cousins, and grandchildren. Our main objective was to identify and study the distribution of genetic risk factors for CJD within these families by applying Mendelian inheritance patterns to systematically quantify the risk percentage of individuals according to carrier status. Consequently, participants were categorized by probabilities of 25%, 50%, and 100% regarding their likelihood of being carriers of the disease. This approach enabled the estimation of the carrier status of participants who opted not to undergo the DNA test for CJD.

Among the 250 participants, only a subset of 35 individuals (14%) were aware of their carrier status, highlighting the ethical and psychological challenges of genetic status awareness in the case of uncurable genetic diseases. This gap in awareness emphasizes the importance of our genetic analysis in providing a clearer picture of the distribution and potential risk of CJD transmission within families. The significance of this analysis can be demonstrated by the fact that 130 participants had an unknown status and a 50% chance of being carriers.

**Table 1:** Demographic and kinship characteristics of gCJD biobank participants

	Carriers				
	Symptomatic Carriers (*N* = 17)	Asymptomatic Carriers (*N* = 35)	Non-Carriers (*N* = 24)	Unknown (*N* = 174)	Total (*N* = 250)
**Age Group (years)**					
< 45	1 (5.9%)	20 (57.1%)	10 (41.7%)	86 (49.4%)	117 (46.8%)
45–65	9 (52.9%)	12 (34.3%)	10 (41.7%)	64 (36.8%)	95 (38.0%)
65–80	7 (41.2%)	3 (8.6%)	2 (8.3%)	20 (11.5%)	32 (12.8%)
> 80	0 (0%)	0 (0%)	2 (8.3%)	4 (2.3%)	6 (2.4%)
**Gender**					
Female	11 (64.7%)	20 (57.1%)	12 (50.0%)	115 (66.1%)	158 (63.2%)
Male	6 (35.3%)	15 (42.9%)	12 (50.0%)	59 (33.9%)	92 (36.8%)
**Likelihood of Being a Carrier**					
100%	17 (100%)	35 (100%)	0 (0%)	0 (0%)	52 (20.8%)
50%	0 (0%)	0 (0%)	0 (0%)	135 (77.6%)	135 (54.0%)
25%	0 (0%)	0 (0%)	0 (0%)	34 (19.5%)	34 (13.6%)
0%	0 (0%)	0 (0%)	24 (100%)	0 (0%)	24 (9.6%)
Missing	0 (0%)	0 (0%)	0 (0%)	5 (2.9%)	5 (2.0%)

## Conclusion

Creutzfeldt–Jakob disease, particularly its genetic variant gCJD, poses a distinctive epidemiological challenge in Israel, accentuated by its prevalence within specific ethnic groups. Understanding the disease’s origins and devising interventions are imperative. The CJD biobank initiative capitalizes on broad collaboration among researchers, physicians, regulators, and the community.

The CJD biobank envisions the future of open collaboration and knowledge exchange, aiming to establish a global community dedicated to CJD reserach. Serving as a hub for researchers worldwide, it offers access to extensive data pools, fostering a collaborative learning environment. Integration with global datasets ensures a comprehensive understanding of the disease.

In conclusion, the roadmap for advancing CJD research in Israel shows promise. By fostering community ties, streamlining regulatory processes, and encouraging open dialogues with researchers and the industry, our initiative aims to revolutionize the comprehension of gCJD and pioneering groundbreaking interventions. We strongly believe that this collaborative model is transferrable to other fields.

## Electronic supplementary material

Below is the link to the electronic supplementary material.

Supplementary Material 1

## Data Availability

The data sets for Fig. 1; Table 1 are available from the CJD Biobank upon request and with appropriate ethical approval in order to preserve inviduals’ privacy.

## Abbreviations

CJD: Creutzfeldt–Jakob disease
gCJD: Genetic Creutzfeldt–Jakob disease
NBB: Negev BioBank
MIDGAM: Israeli National BioBank for Research
GSS: Gerstmann–Sträussler–Scheinker syndrome
FFI: Fatal familial insomnia
HMO: Health Maintenance Organization
SUMC: Soroka University Medical Center
PBMCs: Peripheral blood mononuclear cells
