# Supplementary figures and images for: National Creutzfeldt–Jakob disease research biobank, a novel approach to the establishment of the scientific platform: collaboration between patient advocacy group, scientists, regulators and physicians
Source: Orphanet J Rare Dis. 2025 Apr 10;20:170. doi: 10.1186/s13023-025-03703-6 (PMC11983892; doi:10.1186/s13023-025-03703-6)

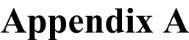


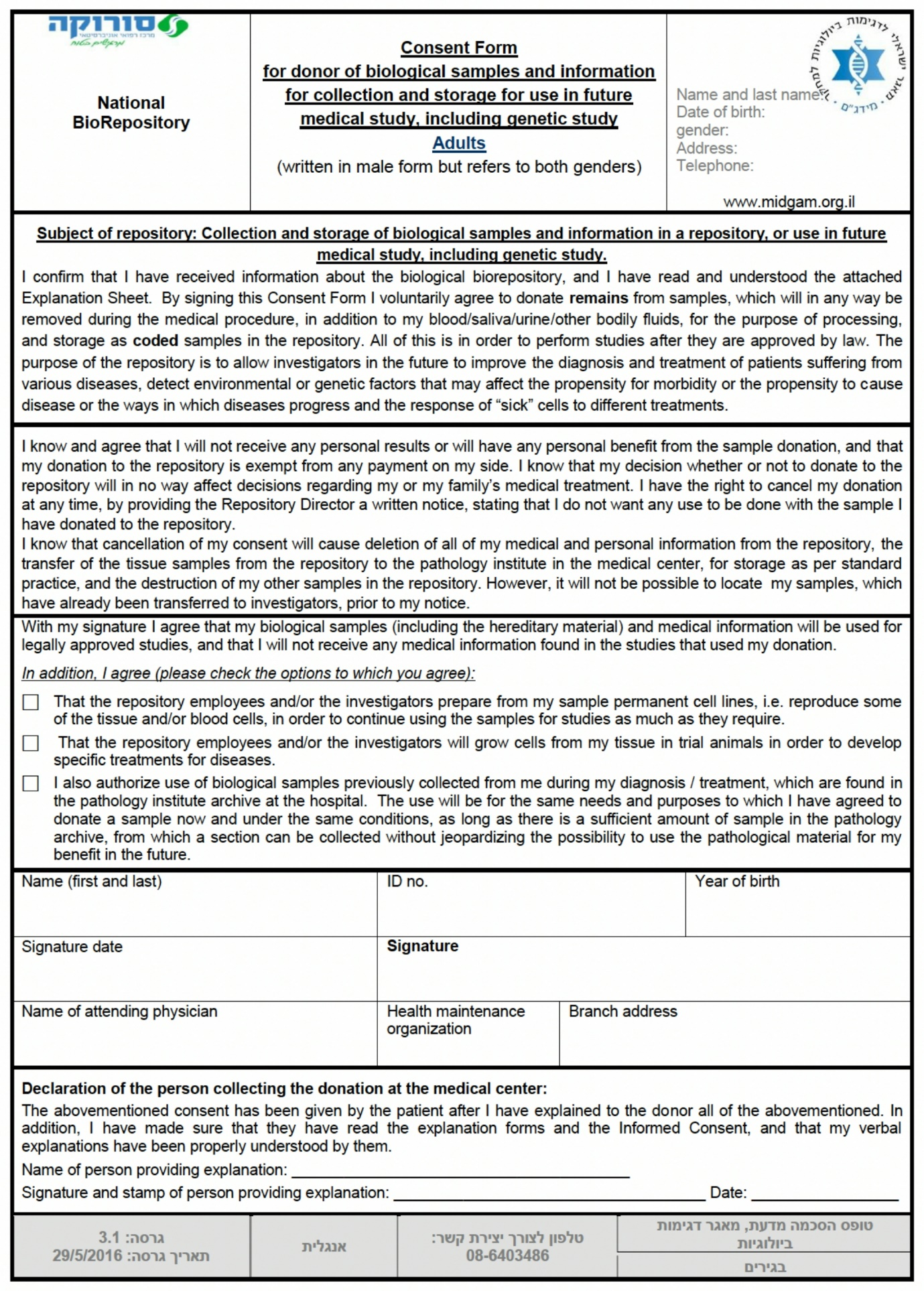


Supplement: Supplementary file 1 — Supplementary Material 1 [file 13023_2025_3703_MOESM1_ESM.docx]
